# Dual-modality treatment using gamma radiation and ZnO nanoparticles: effects on normal and malignant lung cells

**DOI:** 10.1186/s43046-025-00312-z

**Published:** 2025-10-22

**Authors:** Naglaa M. Ismail, Soheir Korraa, Amira Abdel Rehim Qotb

**Affiliations:** 1 Physics Department, Biophysics Division, Faculty of Women for Arts, Science and Education. Ain Shams University, Cairo, Egypt; 2 National Center for Radiation Research and Technology, Atomic Energy Authority, Cairo, Egypt

**Keywords:** Zinc oxide nanoparticles, Gamma radiation, A549 cells, MTT, Cell viability, Apoptosis

## Abstract

This study primarily aims to investigate the effects of gamma (γ) radiation, both independently and in combination with zinc oxide nanoparticles (ZnO NPs), on normal and lung cancer cell lines. Lung cancer continues to be a major cause of cancer-related mortality globally. Radiotherapy is a common way of treating lung cancer. The treatment efficacy of cell death requires a high dosage of focused radiation. Due to their physicochemical properties and potential biological activity, ZnO NPs have emerged as promising candidates in nanomedicine and oncology. In this research, ZnO NPs were synthesized and characterized through various analytical techniques, including X-ray diffraction (XRD), scanning electron microscopy (SEM), transmission electron microscopy (TEM), energy-dispersive X-ray spectroscopy (EDS), differential scanning calorimetry (DSC), and dynamic light scattering (DLS). The resulting nanoparticles were semi-spherical in shape (22–29 nm), stable, and had a zeta potential of – 21 ± 2.40 mV. The cytotoxic effects were assessed using human lung cancer cells (A549) and normal lung fibroblast cells (WI-38). Treatments involved ZnO NPs alone or combined with 15 Gy of γ-radiation over 48 h. A significant increase in cytotoxicity was observed in A549 cancer cells compared to normal cells. ZnO NPs alone showed moderate anticancer efficacy with an IC50 of 26.78 ± 0.44 µg/mL, whereas ZnO NPs + 15 Gy gamma radiation led to a pronounced reduction in cell viability with an IC50 of 15.97 ± 0.45 µg/mL. These results indicate that the combination of ZnO NPs with γ-radiation enhances apoptosis and significantly suppresses the growth of lung cancer cells (*p* < 0.001), offering potential for improved therapeutic outcomes in lung cancer radiotherapy.

## Introduction

Lung cancer remains one of the leading causes of cancer-related mortality worldwide, with approximately 1.8 million new cases diagnosed each year. It is the most fatal cancer among both men and women [1]. Radiotherapy (RT) is a cornerstone in lung cancer treatment and is often employed either alone or in combination with surgery, chemotherapy, or immunotherapy [2]. It is estimated that nearly 60% of all cancer patients undergo radiotherapy at some point during their treatment [3].

Gamma rays, a form of ionizing radiation with low linear energy transfer (LET), are widely used in clinical settings due to their ability to deeply penetrate tissues and induce DNA damage, particularly single and double-strand breaks [4–6]. These breaks can lead to cell cycle arrest and programmed cell death (apoptosis) in malignant cells [7]. However, the therapeutic efficiency of radiotherapy is often limited by factors such as tumor radioresistance and the unintended damage it causes to surrounding healthy tissues [8, 9]. In recent years, the integration of nanotechnology into cancer therapy has shown significant promise in addressing some of these limitations. Nanoparticles, particularly metal-based ones, have demonstrated the ability to enhance the biological effects of radiation by increasing oxidative stress and damaging cellular components [10–12]. Acting as radiosensitizers, these nanomaterials promote the generation of reactive oxygen species (ROS), further amplifying DNA damage in cancer cells while minimizing harm to normal tissues [13]. ZnO NPs possess several advantageous characteristics, including a nanoscale size, tunable electronic properties, and a high surface-area-to-volume ratio, which contribute to their biocompatibility and cost-effective synthesis [14, 15]. In oncology, ZnO NPs have shown selective cytotoxicity by promoting ROS production within tumor microenvironments, leading to mitochondrial dysfunction and cancer cell apoptosis while sparing healthy cells [16–19]. Their use as drug delivery agents and radiosensitizers has further supported their role in enhancing radiotherapeutic outcomes [13, 20].

Importantly, ZnO NPs may act synergistically with gamma radiation by amplifying oxidative stress, locally enhancing radiation-induced DNA damage, and inhibiting repair pathways in cancer cells. This interaction may allow for effective tumor control with lower radiation doses, potentially reducing side effects on normal tissues [21].

Recent studies have also demonstrated that ZnO NPs can sensitize cancer cells to chemotherapy and radiotherapy. For example, their combination with chemotherapeutic agents such as cisplatin and gemcitabine significantly enhanced cytotoxicity in A549 lung cancer cells [22, 23]. Moreover, ZnO NPs have been reported to overcome radiation-induced senescence and resistance by disrupting antioxidant defenses and interfering with DNA repair mechanisms [24, 25]. These findings support the growing interest in ZnO NPs as promising agents to improve cancer treatment outcomes. While most current research is limited to in vitro or preclinical models, the selective action and biosafety of ZnO NPs suggest potential for future clinical translation as adjuncts to radiotherapy. Their integration into clinical protocols could help improve the therapeutic index and minimize radiation-induced toxicity [26].

This study aims to evaluate the combined cytotoxic effects of ZnO NPs and gamma radiation on human lung cancer cells (A549) and normal lung fibroblast cells (WI-38) in vitro. The objective is to assess whether ZnO NPs can selectively enhance the radiosensitivity of cancer cells while minimizing toxicity to normal lung cells, using viability assays and morphological analysis. This evaluation is essential to determine the safety and therapeutic potential of ZnO NPs as radiosensitizers in lung cancer radiotherapy, considering the need to enhance tumor cell kill while minimizing damage to normal tissues [3]. It is hypothesized that combining ZnO NPs and gamma radiation will result in enhanced tumor cell death through increased oxidative stress and DNA damage, with reduced toxicity to normal cells. Recent studies, such as those by [27, 28], further support using ZnO and other metal-based NPs as radiosensitizers in lung cancer models, highlighting their ability to boost treatment outcomes while maintaining safety profiles.

## Materials and methods

### Chemicals and reagents

Analytical-grade reagents were used throughout the study without further purification. Human lung cancer cells (A549) and normal lung fibroblast cells (WI-38) were sourced from the American Type Culture Collection (ATCC, Rockville, MD, USA) and supplied locally through the Regional Center for Mycology and Biotechnology (RCMB) at Al-Azhar University. Cell culture components, including RPMI-1640 medium, l-glutamine, penicillin-streptomycin solution, 0.25% trypsin-EDTA, phosphate-buffered saline (PBS), and fetal bovine serum (FBS), were procured from Lonza (Belgium). Additional reagents such as zinc acetate dihydrate [Zn(CH₃COO)₂·2H₂O], potassium hydroxide (KOH), MTT reagent, dimethyl sulfoxide (DMSO), trypan blue, absolute ethanol (99.9%), as well as apoptosis detection kits containing FITC Annexin V and propidium iodide (PI), were purchased from Sigma-Aldrich (St. Louis, MO, USA).

### Cell culture

Cells were grown in RPMI-1640 medium enriched with 10% heat-inactivated fetal bovine serum (FBS) and 1% penicillin–streptomycin solution. To maintain optimal physiological conditions, the cultures were incubated at 37 °C under a humidified atmosphere containing 5% carbon dioxide. When cells reached approximately 80% confluency, the medium was removed, and the monolayer was gently rinsed with phosphate-buffered saline (PBS, pH 7.4). Cells were then detached by adding 3 mL of 0.25% trypsin–EDTA and incubated briefly at 37 °C until detachment was observed under an inverted microscope. The detached cells were neutralized with fresh medium, centrifuged at 1200 rpm for 5 min, and resuspended in complete RPMI-1640. 1 × 10^4^ cells per well in 96-well plates for viability assays. Cells were incubated for 24 h post-seeding before treatments [29].

### Zinc oxide nanoparticles preparation

Zinc oxide nanoparticles preparation followed [30, 31]. In short, methanol (31.25 mL) was used to dissolve (3.35 mmol) of zinc acetate dihydrate, then methanol (16.25 mL) was used to dissolve potassium hydroxide (6.59 mmol) to create a second solution. At 60 °C, the zinc acetate solution was vigorously stirred while the potassium hydroxide solution was slowly added. The solution turned Murky after the nanoparticles began to precipitate after 1.5 h. After 2 h, the stirrer and heater were discarded, and the mixture was left to settle for two additional hours. After removing the surplus mother Liquor, the precipitate was cleaned with methanol and dried at 60 °C. The concentrations of zinc oxide nanoparticles (3.9–500 µg/mL) used in subsequent experiments were selected based on preliminary cytotoxicity tests and supported by previous studies demonstrating effective dose ranges for ZnO-NPs in cancer cell lines [13, 16, 19].

### Characterization techniques

#### X-ray diffraction (XRD)

The crystalline phases of the prepared ZnO nanoparticles were identified using an X-ray diffractometer (Shimadzu XRD-6000). The measurements were conducted at ambient temperature, scanning across a 2θ angle range from 4° to 90°. The analysis utilized Cu Kα radiation with a wavelength of 0.15408 nm, operated under a voltage of 40 kV and a current of 30 mA. A scan speed of 8° per minute was applied to ensure detailed peak resolution. The diffraction patterns obtained were used to confirm the crystal structure and evaluate the phase purity of the samples [32].

#### High-resolution transmission electron microscopy (HR-TEM)

High-resolution transmission electron microscopy (HR-TEM) was employed to investigate the morphological features and particle size of the synthesized ZnO nanoparticles. Imaging was performed using a JEOL JEM-2100 microscope (Tokyo, Japan), operating at an acceleration voltage of 200 kV. Before analysis, the ZnO nanoparticle suspension was ultrasonicated for 20 min to enhance dispersion and minimize aggregation. A few microliters of the dispersed suspension were drop-cast onto a carbon-coated copper grid and air-dried at room temperature before microscopic examination [33].

#### Dynamic light scattering (DLS)

Dynamic light scattering (DLS) was utilized to determine the size distribution and zeta potential of ZnO nanoparticles. Measurements were carried out using the Nanotrac Wave II system (Microtrac, USA). Samples were dispersed in distilled water, which was selected as the measurement medium due to its influence on colloidal interactions, particle stabilization, and potential aggregation. Before analysis, the nanoparticle suspension was sonicated to ensure proper dispersion [34].

#### Scanning electron microscopy (SEM) and energy dispersive X-ray analysis (EDX)

The surface morphology and the chemical composition of ZnO NPs were characterized by a scanning electron microscope (SEM) (*ZEISS EVO LS 15*). A sputter coater instrument (Quorum Q150R S) must provide an ultrathin layer of electrically conducting gold material to all samples before SEM imaging [35].

#### Differential scanning calorimetry (DSC)

DSC scans were obtained with a DSC-50 Shimadzu scanning calorimeter made in Japan. The samples and reference cells of DSC were measured at room temperature, and the scan was started at (2 °C/min scan rate. The scan ran to 600 °C [36].

### Gamma irradiation

A549 cells were irradiated once with gamma radiation at doses of 5, 10, and 15 Gy, delivered at a rate of 0.675 rad/s using a Gamma Cell-40 irradiator (Nordion, Canada) containing a cesium-137 source. The irradiation system was standardized and calibrated per IAEA guidelines to ensure precise and reproducible dosimetry (IAEA, 2000) [37]. These doses reflect commonly used exposures in in vitro radiotherapy studies to evaluate dose-dependent cellular responses relevant to clinical radiotherapy practice [38].

### Cytotoxic activity using MTT assay

The cytotoxic effects of the test materials on A549 and WI-38 cell lines were evaluated using the MTT assay, following the method described by [39] with slight modifications. Briefly, cells were seeded into 96-well plates at a density of 5 × 10^4^ cells/well and incubated for 24 h at 37 °C in a 5% CO₂ atmosphere to allow cell attachment. After treatment with various concentrations of zinc oxide nanoparticles, without or by exposure to different dosages of γ-radiation (5, 10, and 15 Gy) for 48 h, 20 µL of MTT solution (5 mg/mL in PBS) was added to each well, and the plates were incubated for an additional 4 h. Following incubation, the medium was carefully removed, and 150 µL of DMSO was added to each well to dissolve the formazan crystals formed by viable cells. The absorbance was measured at 570 nm using a microplate reader. Cell viability was calculated as a percentage relative to untreated control cells using the formula:

$$Cellviability\;\left(\%\right)=(OD_{570}oftreatedcells/OD_{570}ofcontrolcells)\times100$$

All experiments were performed in triplicate, and results were expressed as mean ± standard deviation.

### Cell apoptosis assay

To investigate the mode of cell death induced by zinc oxide nanoparticles (ZnO-NPs), A549 cells were treated with the IC₅₀ concentration of ZnO-NPs alone or in combination with 15 Gy of γ-radiation for 48 h. Apoptosis was evaluated using a FITC Annexin V/P Propidium Iodide (PI) detection kit (Sigma-Aldrich, St. Louis, MO, USA), following the manufacturer’s instructions and standard flow cytometric protocols [40]. After treatment, cells were harvested, washed twice with cold phosphate-buffered saline (PBS), and resuspended in 100 µL of binding buffer. Subsequently, 5 µL of Annexin V-FITC and 5 µL of PI were added to each sample, followed by incubation in the dark for 15 min at room temperature. After staining, 400 µL of 1× binding buffer was added, and the samples were incubated for an additional 20 min in the dark before analysis. Apoptotic profiles were determined using a BD Accuri™ C6 Plus flow cytometer by acquiring 50,000 events per sample. Data were analyzed to distinguish between four populations: viable cells (Annexin V⁻/PI⁻), early apoptotic cells (Annexin V⁺/PI⁻), late apoptotic cells (Annexin V⁺/PI⁺), and necrotic cells (Annexin V⁻/PI⁺).

### Statistical analysis

All experimental procedures were carried out in technical triplicate using the same assay plate to ensure consistency. Data are reported as the mean ± standard deviation (SD). Statistical comparisons between groups were performed using one-way analysis of variance (ANOVA), followed by Tukey’s multiple comparison test. Analyses were conducted using GraphPad Prism software (version 5.0, GraphPad Software Inc., San Diego, CA, USA). A *p*-value less than 0.001 was considered statistically significant.

## Results and discussion

### X-ray diffraction (XRD)

**Fig. 1:**
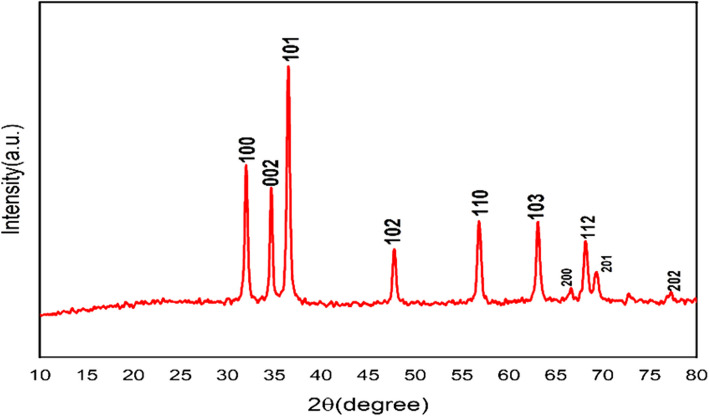
XRD pattern of ZnO NPs

X-ray diffraction analysis provides insights into the crystal structure of the samples. The XRD pattern of ZnO nanoparticles is depicted in Fig. 1. The diffraction peaks for ZnO nanoparticles were more intense and narrower, indicating that these nanostructures exhibit good crystallinity. The peaks observed at scattering angles (2θ) of 31.98°, 34.66°, 36.48°, 47.78°, 56.78°, 63.08°, 66.6°, 68.14°, 69.3°, and 77.08° correspond to the (100), (002), (101), (102), (110), (103), (200), (112), (201), and (202) crystal planes, respectively [41].

### Transmission electron microscopy (TEM)

**Fig. 2:**
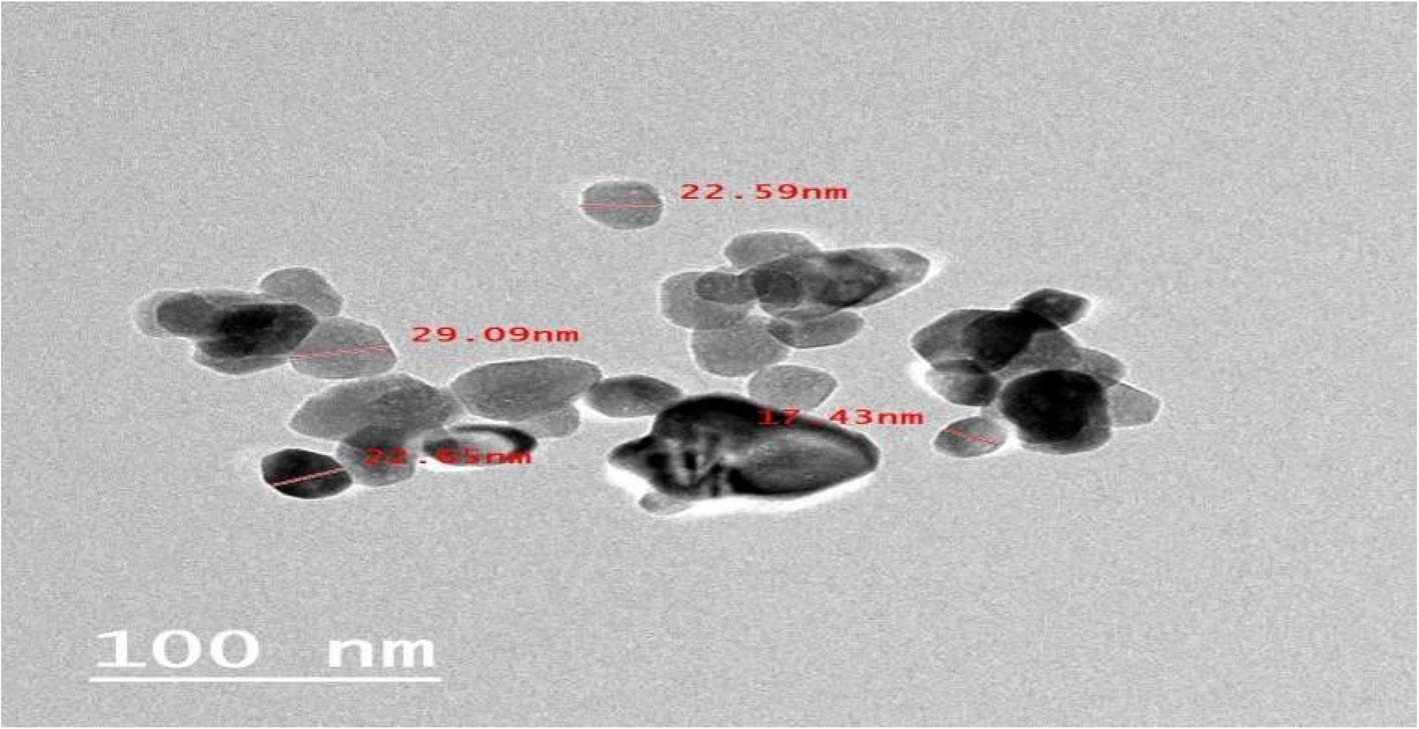
HR-TEM of ZnO NPs

A supplementary morphological description is obtained using the (HR-TEM) analysis. The (HR-TEM) image of ZnO NPs with a mean size of 22.94 ± 4.77 nm is shown in Fig. 2. The image demonstrates that the particles are spherical [42].

### Dynamic light scattering (DLS)

**Fig. 3:**
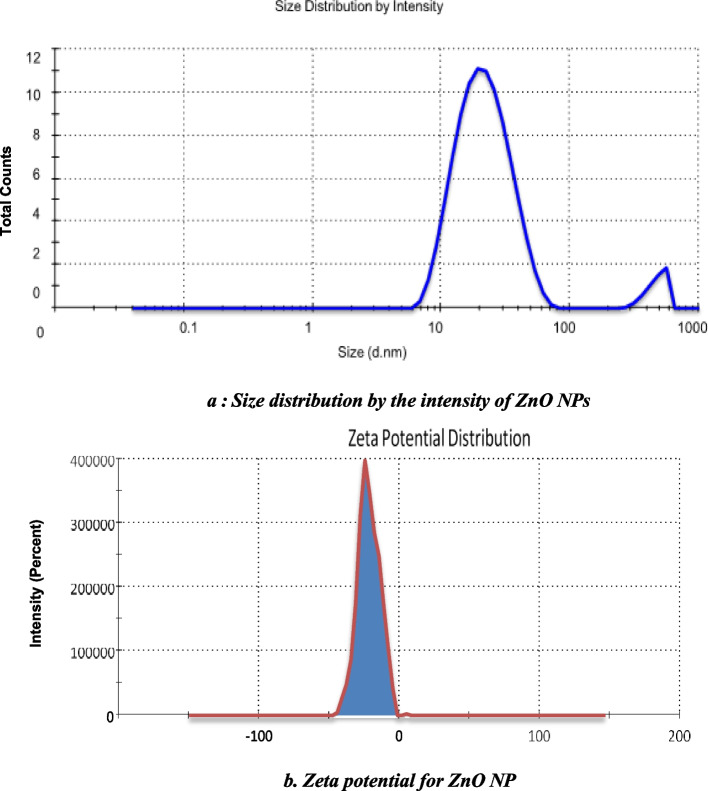
**a** Size distribution by the intensity of ZnO NPs. **b** Zeta potential for ZnO NP

The particle size and zeta potential of the synthesized ZnO nanoparticles are presented in Fig. 3a, b, respectively. The mean particle size was 24 ± 3.06 nm, with a narrow distribution as indicated by a low polydispersity index (PDI) of 0.324. Zeta potential measurements were conducted to evaluate the surface charge and colloidal stability of the ZnO-NPs. The average zeta potential was – 21 ± 2.40 mV, indicating a moderately negative surface charge (Fig. 3b). This level of surface charge is generally associated with good dispersion stability in aqueous environments [34, 43]. It may also influence cellular interactions, potentially enhancing cellular uptake and selective internalization in cancer cells due to electrostatic interactions with the cell membrane [44]. These properties are important when considering the biological behavior and cytotoxic effects of nanoparticles in subsequent assays. In comparison with previous studies, similar zeta potential values ranging from − 20 to −30 mV have been linked to enhanced nanoparticle uptake and biological activity in various cancer models. For example, ZnO nanoparticles with a zeta potential of approximately − 22.4 mV showed increased cytotoxicity in HepG2 cells [43], while those with a surface charge near − 25.7 mV exhibited improved internalization in MCF-7 breast cancer cells [45]. Such zeta potential values are generally considered optimal for promoting cellular uptake while maintaining colloidal stability, suggesting that the zeta potential measured in the present study falls within an effective range for biological applications.

### Scanning electron microscopy (SEM) and energy dispersive X-ray analysis (EDX)

**Fig. 4:**
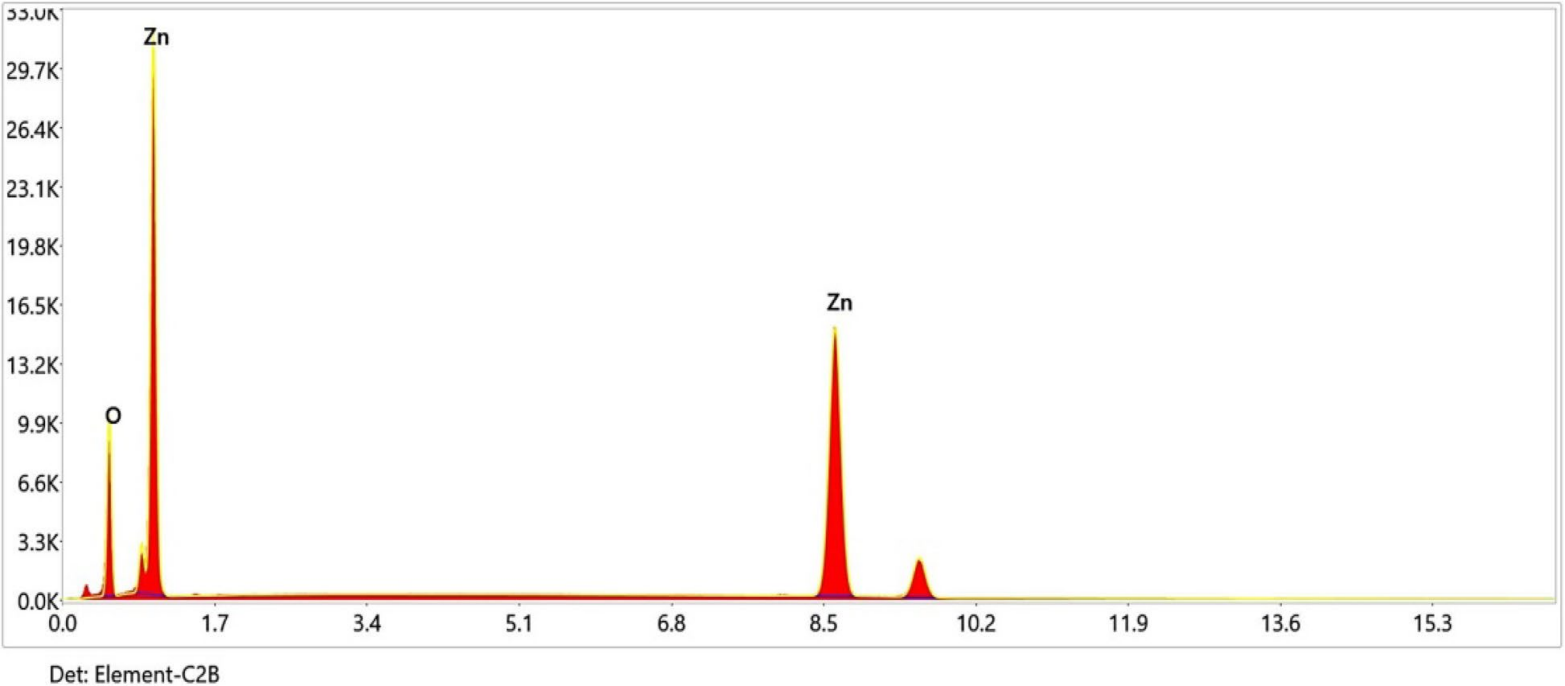
EDX of ZnO NPs

**Fig. 5:**
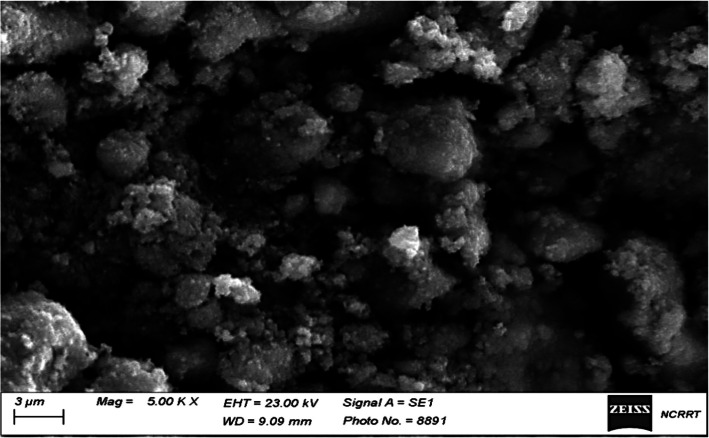
SEM of ZnO NPs

SEM images were used to examine the surface morphology of ZnO nanoparticles, as shown in Fig. 5. The findings demonstrated the spherical form of ZnO NPs. The elemental composition of ZnO NPs was determined by EDX analysis. Agglomeration is responsible for the appearance of some larger ZnO NPs in SEM scans [46]. Figure 4 displays the EDX spectra of samples of ZnO nanoparticles. The label for the ZnO sample displays the element names and percentages. Since zinc and oxygen are the sample’s primary ingredients [47], no traces of contaminants were detected within the EDX detection limit. Figure 5 and Table 1 illustrate the weight percentages of ZnO NPs, which were 75.64% Zn and 24.36% O.

**Table 1:** The EDX of ZnO NPs

Materials	Element	Weight %	Atomic %	Net Int	Error %
ZnO	O	24.36	56.83	1008.02	10.09
	Zn	75.64	43.17	4161.57	1.84
Total		100	100		

### Differential scanning calorimetry (DSC)

**Fig. 6:**
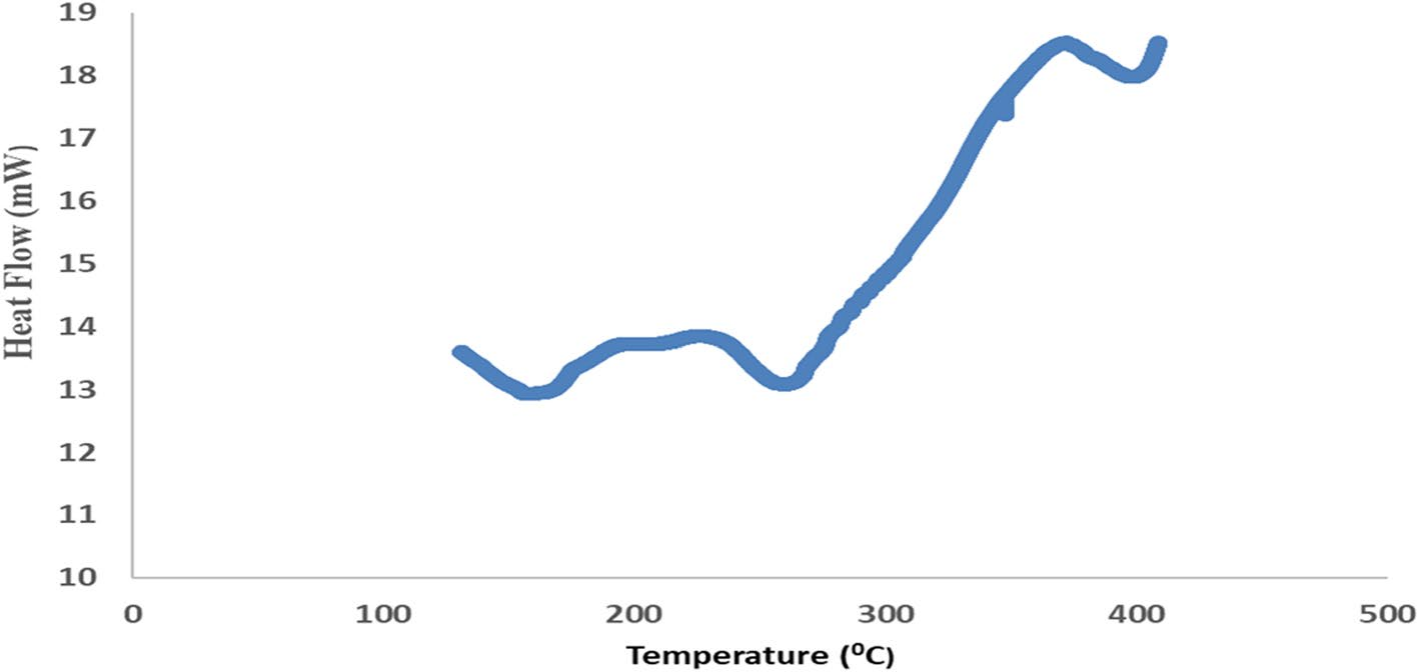
DSC thermogram of synthesized ZnO nanoparticles

The DSC technique was employed to investigate the oxidized structure and isothermal oxidation behavior of ZnO nanoparticles in ambient air at temperatures ranging from 50 to 600 °C. The DSC curves of ZnO nanoparticles are displayed in Fig. 6. The low-temperature endothermic peak at 155.7 °C can be attributed to the loss of volatile surfactant molecules adsorbed on the surface of ZnO nanoparticles during synthesis. A second thermal event near 261.2 °C can be attributed to the decomposition of intermediate zinc hydroxide phases, leading to the formation of ZnO nanoparticles. A broader endothermic peak at approximately 401.2 °C may result from internal structural rearrangements or the elimination of residual organic content. No indication of ZnO reduction to metallic zinc is observed under these oxidative conditions, as ZnO remains thermally stable in air at these temperatures [48].

### Cytotoxic activity on (A549) and (WI-38) by MTT assay

**Fig. 7:**
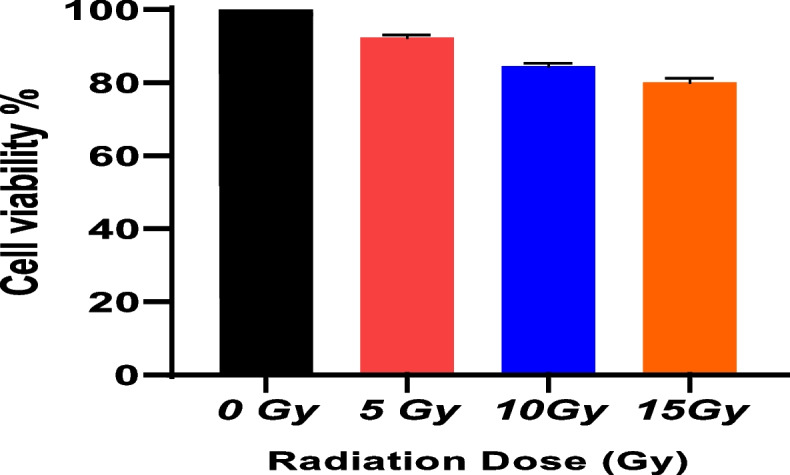
The cytotoxic effect of different doses of gamma radiation 5, 10, 15 Gy, on the A549 cell Line for 48 h

The A549 lung cancer cell Line demonstrated considerable resistance to gamma radiation at 5 to 15 Gy doses. Even at the highest dose of 15 Gy, cell viability remained above 70%, indicating that the rate of cell death did not exceed 30% (Fig. 7). While 5 Gy of gamma radiation induced noticeable toxicity in A549 cells, its cytotoxic impact was less pronounced than 10 and 15 Gy doses. This resistance implies that A549 cells possess efficient cellular mechanisms that protect against radiation-induced damage and enable recovery [49, 50]. Viability assessments conducted 48 h post-irradiation showed sustained metabolic activity in A549 cells, with survival rates frequently exceeding 80% even after exposure to 15 Gy. Clonogenic assays corroborated these findings by revealing that A549 cells are less susceptible to radiation-induced reproductive death compared to other, more radiosensitive lung cancer lines [51]. This inherent radioresistance is largely attributed to proficient DNA repair systems. Upon exposure to ionizing radiation, A549 cells rapidly initiate pathways to repair double-strand breaks (DSBs), a critical form of lethal DNA damage caused by radiation [49, 50]. Additionally, radiation generates reactive oxygen species (ROS) that inflict oxidative damage on cellular components, including DNA and lipids [52]. Gamma radiation remains a cornerstone in lung cancer radiotherapy by inducing DNA lesions that trigger cancer cell death; however, its efficacy is often compromised by the tumor cells’ radioresistance and collateral damage to healthy lung tissue [53]. Our findings confirm that higher radiation doses amplify the toxic effects of ZnO nanoparticles on lung cancer cells, highlighting their potential as radiosensitizers to improve radiotherapy outcomes. This strategy could also enable reduced radiation doses, minimizing harm to normal tissues while maintaining therapeutic efficacy.

**Fig. 8:**
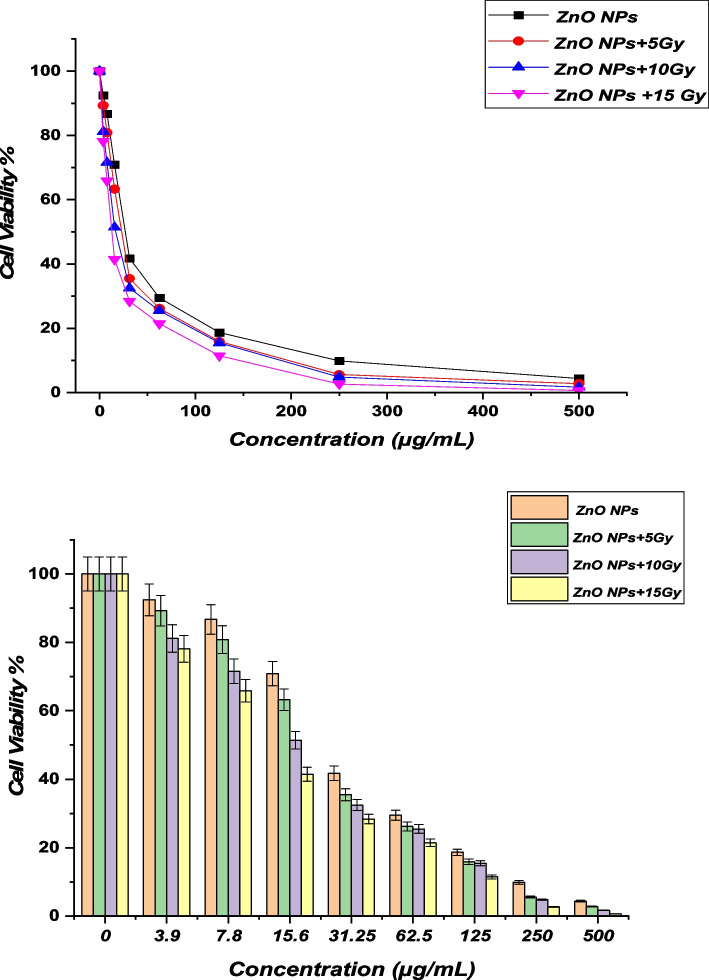
The cytotoxic effect of different concentrations of ZnO NPs combined with different doses of radiation, 5, 10, 15 Gy (γ-radiation) on the A549 cell Line for 48 h

When A549 cell lines were exposed to various concentrations of ZnO nanoparticles (ZnO NPs) combined with different γ-radiation doses (5, 10, and 15 Gy), a significant, dose-dependent decrease in cell viability was observed. This effect was more pronounced in the combination treatment compared to ZnO NPs or γ-radiation alone after 48 h, as illustrated in Fig. 8.

**Fig. 9:**
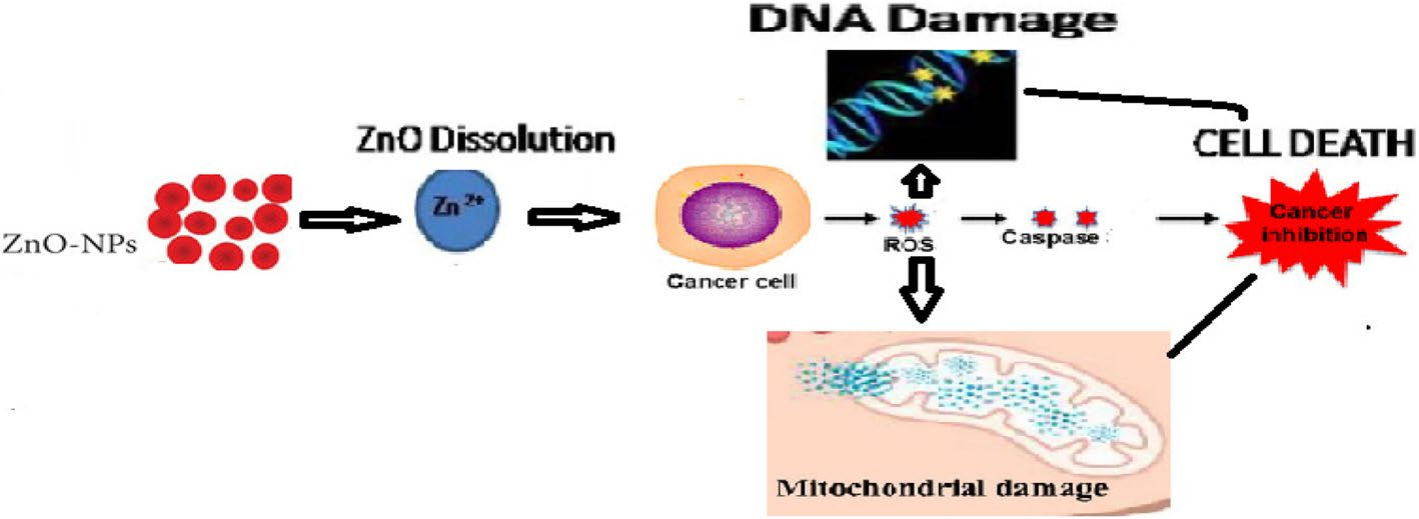
A schematic mechanism by which nano zinc oxide can potentially cause cancer cells to become toxic or undergo apoptosis

Our findings demonstrate that the IC₅₀ value for ZnO NPs alone was 26.78 ± 0.44 μg/mL, which is consistent with earlier studies reporting IC₅₀ values between 25 and 30 μg/mL for A549 cells under similar in vitro conditions**.** However, this value dropped significantly to 17.97 ± 0.45 μg/mL upon combining ZnO NPs with 15 Gy γ-radiation, indicating a clear synergistic effect (*p* < 0.001). This enhanced cytotoxicity is likely driven by an increase in reactive oxygen species (ROS) generated by both ZnO NPs and γ-radiation [54, 55]. This represents a 32.8% reduction in IC₅₀, which appears more substantial than effects reported in other cancer cell types. For comparison, studies in PC-3 prostate cancer cells showed only a 20% reduction in IC₅₀ (from 28.3 to 22.6 μg/mL) when combining ZnO NPs with 10 Gy radiation [56]. Similar cytotoxic responses to ZnO NPs have been documented in various cancer cell types, including MCF-7 breast cancer cells (IC₅₀ of 27.3 μg/mL) [57], HeLa cervical cancer cells (~ 30.1 μg/mL) [58], and HepG2 liver cancer cells (~25.6 μg/mL) [59]. While less data is available on combination treatments with radiation, the pronounced reduction in IC₅₀ observed in this study highlights the potential of ZnO NPs to act as radiosensitizers across different tumor models (Fig. 9).

ROS accumulation leads to oxidative stress that damages DNA, proteins, and mitochondria, ultimately triggering apoptosis. Cancer cells, including A549, are particularly sensitive to oxidative stress due to their high metabolic rates and deficient antioxidant defenses [19, 60, 61]. Gamma radiation further amplifies ROS production through water radiolysis [61], and ZnO NPs are known for their photocatalytic properties that intensify ROS formation under irradiation [62, 63]. Similar synergistic effects have been reported in other studies where ZnO NPs enhanced radiation-induced cytotoxicity in lung cancer cells [23, 64]. Some studies suggest that ZnO NPs may impair DNA repair pathways in cancer cells, particularly after radiation-induced damage. By interfering with repair mechanisms such as homologous recombination or non-homologous end joining, ZnO NPs may promote the accumulation of unrepaired DNA breaks, pushing cells toward apoptosis. This potential mechanism is supported by findings in which ZnO NPs enhanced the effect of chemotherapy agents and reduced radioresistance in A549 cells by disrupting redox balance and inhibiting cellular recovery after stress [21, 22, 24]. Altogether, these findings reinforce the potential of ZnO NPs as effective radiosensitizers. Their combination with γ-radiation not only increases cancer cell death but may also allow for the use of lower radiation doses, thus minimizing adverse effects on surrounding healthy tissues. Further in vivo and mechanistic studies are warranted to explore long-term safety and therapeutic potential.

**Fig. 10:**
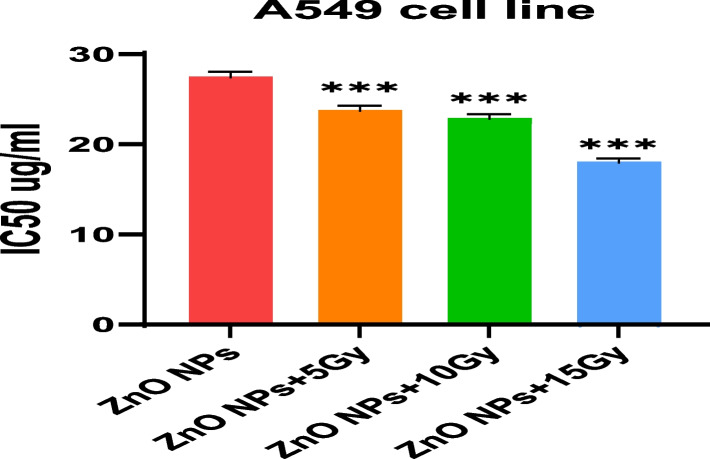
IC50 values of ZnO NPs with various doses of γ-radiation on the A549 cell line. ***p value < 0.001 refers to that there is a statistically significant difference as compared to ZnO NPs treatment

The results presented in Table 2 and Fig. 10 provide the half maximal inhibitory concentration (IC50 μg/mL) values of ZnO NPs in combination with different doses of γ-radiation.

**Table 2:** IC50 values (μg/mL) of ZnO NPs on human cancer cell line (A549) and in combination with different doses of γ radiation after 48 h

IC50(μg/mL) A549 cells				
Compound	Without radiation	With radiation		
		5 Gy	10 Gy	15 Gy
ZnO NPs	26.78 ± 0.44	23.04 ± 0.58	22.32 ± 0.49	17.97 ± 0.45

**Fig. 11:**
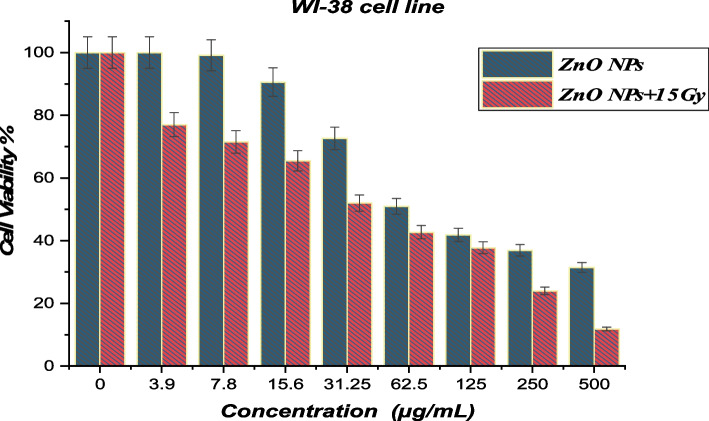
The cytotoxic effect of different concentrations of ZnO NPs alone or combined with 15 Gy (γ-radiation) on the WI-38 cells for 48 h

In the current study, the cytotoxic effects of zinc oxide nanoparticles (ZnO NPs) on normal lung fibroblast cells (WI-38) were evaluated both independently and in combination with gamma radiation as illustrated in fig. 11. The findings revealed a clear concentration-dependent reduction in cell viability, with higher concentrations (500 μg/mL) reducing viability to approximately 31% (*p* < 0.001) compared to control). While lower doses did not induce significant cytotoxicity (*p* > 0.001), the threshold for toxicity appears to intensify beyond 31.2 μg/mL, where cell viability was significantly decreased (*p* < 0.001). Notably, when ZnO NPs were combined with 15 Gy of gamma radiation, cell viability decreased even further, showing a statistically significant reduction compared to either treatment alone (*p* < 0.001). This synergistic effect was quantitatively reflected in a significant decrease in the half-maximal inhibitory concentration (IC₅₀) from 81.91 ± 0.96 μg/mL (without radiation) to 49.88 ± 0.65 μg/mL under irradiation (*p* < 0.001), as shown in Table 3. These observations align with several prior investigations demonstrating the ability of nanoparticles to sensitize cells to radiation. ZnO NPs, in particular, have been shown to elevate radiosensitivity through mechanisms involving enhanced oxidative stress leading to DNA damage and disruption of repair processes [65–67]*.* Moreover, the combined exposure to ZnO NPs and ionizing radiation has been linked to increased genetic damage and programmed cell death in both malignant and healthy cell lines, corroborating the mechanisms suggested by our findings [68–71]*.* Additionally, [72] showed that green-synthesized tin nanoparticles exhibited strong biological activity, including anti-haemolytic effects, underscoring the therapeutic potential of eco-friendly nanoparticle production for biomedical uses. Their results support the idea that nanoparticles, beyond targeting cancer, may also provide protective or restorative functions in biological systems, which is crucial when considering toxicity to normal cells. Supporting these insights, [73] found that silver nanoparticles made from Anethum graveolens extract significantly boosted radiotherapy effectiveness in colon cancer models, illustrating the benefits of combining metal nanoparticles with natural extracts. Similarly, [74] created iron-based nanoparticles that improved chemotherapy delivery and efficacy against lung cancer cells, demonstrating the multifunctionality of such nanosystems. [75] also showed that green-synthesized Cu₂O nanoparticles from Camellia sinensis leaves have notable antioxidant and anticancer activity, highlighting the advantages of eco-friendly synthesis methods. In addition, [76] developed composite nanofibers made of polyvinyl alcohol, gum tragacanth, and graphene oxide, which enabled targeted antibiotic release, exemplifying nanomaterials’ versatility in drug delivery. Likewise, [77] demonstrated that silver nanoparticles coated on chitosan-alginate magnetite not only catalyzed chemical reactions but also protected lung cells, indicating a combined therapeutic and protective effect of nanoparticle formulations.

**Table 3:** IC50 values (µg/mL) of ZnO NPs on normal lung cells (WI-38) and in combination with 15 Gy of γ-radiation after 48 h

IC50(μg/mL) WI-38 cells		
Compound	Without radiation	With radiation
		15 Gy
ZnO NPs	81.91 ± 0.96	49.88 ± 0.65

Despite promising therapeutic implications, the observed cytotoxicity in normal lung fibroblast cells highlights the necessity for carefully delineating safe dosing parameters to minimize damage to healthy tissues. Additionally, it should be noted that the single high dose of gamma radiation (15 Gy) used in this study differs from the fractionated dosing regimens typically employed in clinical radiotherapy. Therefore, subsequent studies should focus on fractionated radiation schedules combined with ZnO NPs to better replicate clinical conditions and optimize the therapeutic index.

However, it is important to note that the single high radiation dose (15 Gy) used in this study does not reflect standard clinical radiotherapy practices. Future experiments should incorporate fractionated dosing schemes to more effectively assess radiosensitizing effects under clinically relevant conditions. While a single supra-clinical dose of 15 Gy was used in this study, we acknowledge that a fractionated dosing regimen (e.g., 2 Gy × 5 fractions) may better reflect clinical radiotherapy practices. Future studies should explore the effects of such fractionated protocols to enhance clinical relevance [78] (Fig. 11).

### Apoptosis analysis by flow cytometry

**Fig. 12:**
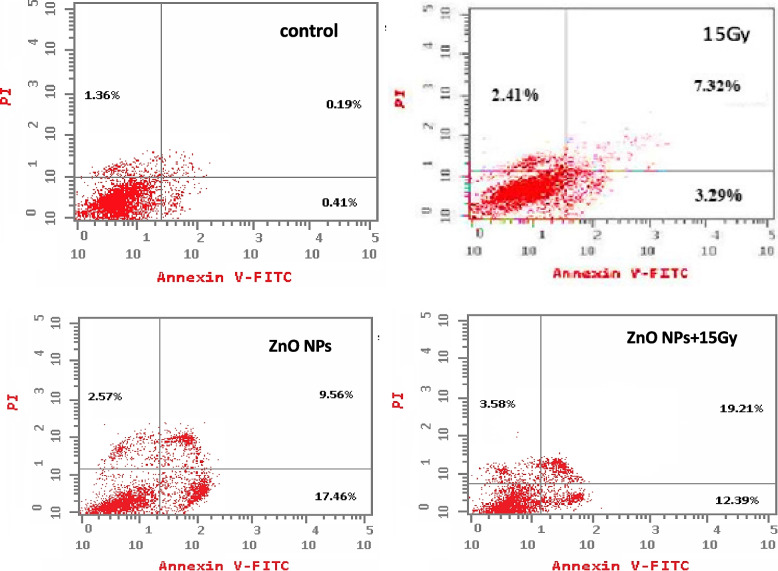
Apoptosis induction by ZnO NPs treatment combined with or without 15 Gy of γ-radiation after 48 h on the A549 cell line

This work analyzed the cellular apoptosis and necrosis of ZnO NPs on lung cancer cell line (A549) without or with gamma radiation at a dose of 15 Gy for 48 h using flow cytometry with an annexin V/PI double staining. The treatment with the IC50 (μg/mL) concentration of ZnO NPs increases apoptosis percentages in A549 cells as compared to the untreated cells. However, compared to ZnO NPs or gamma radiation alone, the rates of apoptosis in the combination of ZnO NPs and 15 Gy of gamma radiation were noticeably higher Fig. 12 and Table 4.

**Table 4:** Assessment of A549 cell apoptosis following 48 h of ZnO NPs applied alone or in combination with 15 Gy

Samples	Cell viability	Apoptosis			Necrosis
		Total	Early	Late	
Control A549	98.04	1.96	0.41	0.19	1.36
A549 + 15 Gy	86.98	13.02	3.29	7.32	2.41
ZnO NPs	70.41	29.59	17.46	9.56	2.57
ZnO NPs + 15 Gy	64.82	35.18	12.39	19.21	3.58

The present study investigated the anticancer efficacy of zinc oxide nanoparticles (ZnO NPs), both alone and in combination with gamma (γ) radiation, against A549 lung carcinoma cells. ZnO NPs have garnered considerable interest in cancer therapy due to their ability to generate reactive oxygen species (ROS), particularly when activated by ionizing radiation. This leads to elevated oxidative stress, DNA damage, and ultimately, the induction of apoptosis in cancer cells. The radiosensitizing effect of ZnO NPs is mainly attributed to the catalytic activity of their surfaces under radiation, which amplifies oxidative stress levels within tumor cells.

As a result of these mechanisms, ZnO NPs activate apoptosis pathways in cancer cells. In particular, nano ZnO mediates ROS production through the p53 pathway. The p53 protein becomes activated in response to oxidative stress–induced DNA damage, thereby initiating programmed cell death [64].

The findings of the present study align with these mechanisms. ZnO NPs exhibited notable cytotoxicity against the A549 lung carcinoma cell line. Furthermore, when combined with gamma (γ) radiation, a synergistic cytotoxic effect was observed. This combined treatment led to a significant reduction in cell viability and a pronounced increase in apoptosis compared to either treatment alone [68].

Furthermore, supporting our findings, earlier research demonstrated that ZnO nanoparticles induce apoptosis in various cancer cell Lines. Notably, one study reported approximately 40% total apoptosis in MCF-7 breast cancer cells after 48 h of treatment with ZnO NPs [79]. Similarly, around 38% apoptosis was observed in HepG2 liver cancer cells through ROS-mediated mitochondrial pathways [80]. Comparable apoptotic effects (~ 36%) were also reported in A549 lung carcinoma cells [81]. Our findings of 29.6% total apoptosis in A549 cells treated with ZnO NPs alone fall within this range, supporting the broad cytotoxic potential of ZnO nanoparticles across different cancer types.

Additionally, it is crucial to highlight that both apoptosis and necrosis can result from elevated intracellular Zn^2^⁺ levels. Numerous studies have indicated that Zn^2^⁺ increases occur due to the dissolution of ZnO nanoparticles (NPs) in lysosomes after cellular uptake, or when ZnO NPs dissolve in the extracellular space, facilitating the transport of Zn^2^⁺ ions into the cell. This leads to a rise in intracellular Zn^2^⁺ concentrations that surpasses the capabilities of the Zn^2^⁺ homeostatic system. Consequently, the toxic levels of Zn^2^⁺ disrupt the mitochondrial membrane potential, prompting the generation of reactive oxygen species (ROS) and DNA fragmentation, activating caspases and resulting in apoptosis. When Zn^2^⁺ concentrations escalate further, necrosis becomes the predominant mode of cell death [70].

## Conclusion

Zinc oxide nanoparticles (ZnO NPs) demonstrated potent cytotoxic activity against lung cancer cells, particularly when used in combination with gamma radiation. This enhanced effect is primarily attributed to increased production of reactive oxygen species (ROS), which induce oxidative stress, DNA damage, and apoptosis in malignant cells. The observed synergistic interaction suggests that ZnO NPs may sensitize cancer cells to ionizing radiation, offering a strategy to improve the effectiveness of radiotherapy and potentially overcome tumor radioresistance. Importantly, while ZnO NPs also affected normal lung fibroblast cells (WI-38) at higher concentrations, their cytotoxicity remained significantly lower compared to cancer cells, indicating a favorable therapeutic window. This selectivity underscores the importance of dose optimization to maximize anticancer efficacy while minimizing potential toxicity to healthy tissue.

In conclusion, the integration of ZnO NPs with conventional radiotherapy presents a promising avenue for developing more effective and targeted lung cancer treatments. Further in vivo studies and clinical evaluation are warranted to validate these findings and assess their translational potential.

### Study limitations 

This research has certain limitations that should be considered when interpreting the results. Firstly, the experiments were conducted using only one lung cancer cell line (A549), which may not fully reflect the diversity of cancer cell behavior. Secondly, although the effects of radiation were assessed on these cancer cells, normal lung fibroblast cells (WI-38) were not exposed to radiation alone. This limits the ability to evaluate the selectivity of the treatment directly. Thirdly, all experiments were performed under in vitro conditions, which do not fully replicate the complex biological environment of living organisms; therefore, in vivo studies are necessary to validate these findings. Additionally, apoptosis was primarily assessed through morphological observations and Annexin V/PI staining, without confirmation by specific molecular markers such as caspase-3 and caspase-9 activity assays. Finally, although reactive oxygen species (ROS) generation was proposed as a key mechanism underlying treatment efficacy, direct measurement of ROS was not performed. These points highlight the need for further investigations to support and expand on the current findings.

## Data Availability

No datasets were generated or analysed during the current study.
